# Tricho-rhino-phalangeal syndrome - clinical, trichoscopic and radiological images^☆^

**DOI:** 10.1016/j.abd.2021.09.018

**Published:** 2022-11-04

**Authors:** Evelyn Freitas Rodrigues, Lisa Gava Baeninger, Caroline Romanelli

**Affiliations:** Hospital e Maternidade Celso Pierro, Pontifícia Universidade Católica de Campinas, Campinas, SP, Brazil

Dear Editor,

Tricho-rhino-phalangeal syndrome (TRPS) type I is a rare condition first described by Giedion in 1966. The main characteristics are sparse and slow-growing hair, a pear-shaped nose and coned epiphyses on the medial phalanges of the hands. The hair shafts are thin and miniaturized, as in androgenetic alopecia. There is a down-regulation of the TRPS1 gene in the baldness area, and the decrease of the same protein can impair endochondral cartilage differentiation and cell interactions in the development of hair follicles.^1^

Short stature, Legg-Calve-Perthes Disease (aseptic necrosis of the femoral head), shortening of the toes (clinobrachydactyly), dystrophic nails, long philtrum, thin upper lip, and thinning of the distal third of the eyebrows may occur.^1^, ^2^

TRPS type I often shows an autosomal dominant pattern inheritance, but autosomal recessive inheritance can occur. Type II occurs sporadically, associated with mental retardation and multiple exostoses.^2^

The aim of this study is to report the investigation of a case of TRPS in an eleven-year-old girl with mechanical arthralgia in the knees for three years, without morning stiffness, she presented also shortening of the toes and ulnar deviation of the second, third and fifth fingers, bilateral osteochondromas in the supracondylar region, hair rarefaction, and three episodes of convulsive crisis. The neuropsychomotor development was normal. Parents were non-consanguineous and there was no history of repeated abortions. Regarding family history, the father is being treated for epilepsy, and siblings do not have any signs suggestive of genetic syndromes.

On physical examination, bilateral hypermobility and deformity of the interphalangeal joints, thickening of the wrists, thinning of the skull, collapse of the nasal bridge, thin upper lip, pear-shaped nose (Fig. 1), and high-arched palate were observed. The thorax was short, with a winged scapula. The weight was 29.6 kg, height 141.5 cm (normal and age-appropriate weight and height), with Tanner stage M2P1, menarche at age 11. Trichology showed fine short hair; and diffuse hair rarefaction, more evident in the bitemporal region (Fig. 1C). The pull and tug tests were negative. Analysis of the hair strands under optical microscopy was carried out and the trichogram disclosed normal hair shafts (Fig. 2A). At the highest magnification, normal telogen hairs were disclosed (Fig. 2B). Trichoscopy was normal, but showed fine hairs, corresponding to hypotrichosis (Fig. 2C).

**Figure 1:**
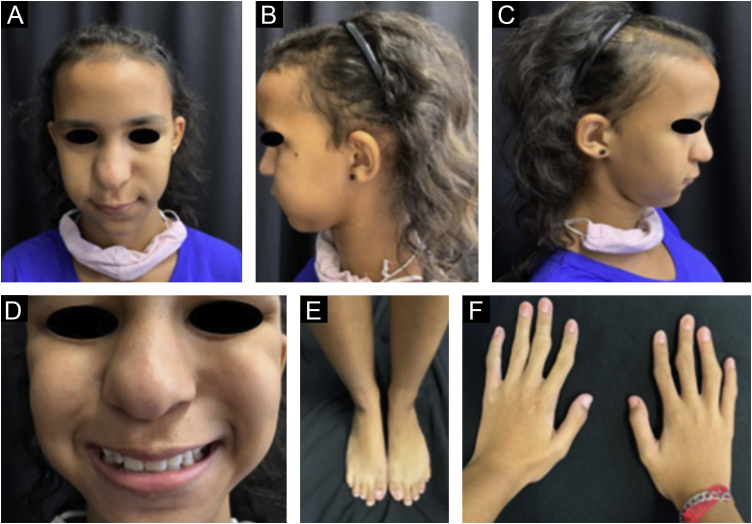
Patient’s (B and C) phenotypic characteristics (A). Pear-shaped nose, diffuse hair rarefaction, more intense in the frontotemporal regions. Close up view of the pear-shaped nose (D). Joint deformities in the feet and hands (E and F).

**Figure 2:**
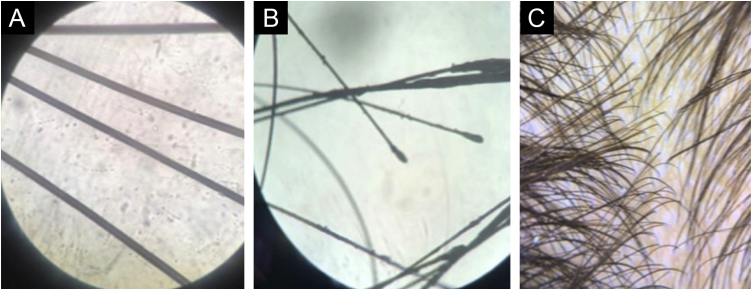
Trichogram. (A) Trichoscopic analysis under optical microscopy showing normal hair shafts. (B) At the highest magnification, normal telogen hairs are observed. (C) Trichoscopy was normal, although it showed fine hairs corresponding to hypotrichosis.

The cranial tomography showed no changes. The knee radiography revealed a well-defined sclerotic lesion along the medial cortex of the distal diaphysis of the right femur. The patient is undergoing clinical follow-up with the Orthopedics team. The hand radiography showed a widening of the base of the middle phalanx from the second to fifth fingers, bilaterally (Fig. 3). The ophthalmologic study and the transthoracic echocardiogram were normal. Of the serological tests, only ANA was positive, with a titer of 1:80 and a speckled nuclear pattern.

**Figure 3:**
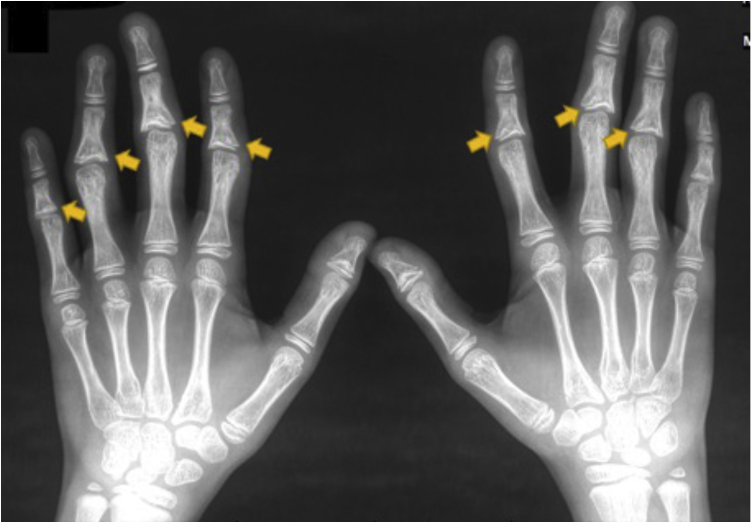
Hand x-ray showing coned epiphyses in the middle phalanges (yellow arrows).

The diagnosis of TRPS type I is attained through typical clinical findings (facial features, ectodermal manifestations such as alopecia, supernumerary teeth, and limb anomalies) and radiographic findings of coned epiphyses, or the identification of a heterozygous pathogenic variant of TRPS1. For type II, the diagnosis is attained through typical findings of TRPS II and a contiguous chromosome 8 deletion that includes the genes TRPS1, RAD21 or EXT1.^3^ Molecular genetic testing is of greater value if the presentation is mild or atypical, otherwise, they are not always necessary for the diagnosis.^4^

Treatment includes supportive measures from a multidisciplinary team (orthopedics, physical therapy, psychology, and medical genetics).^4^ Seizures have been described exclusively in association with KCNQ3 deletion, in addition to TRPS1 and EXT1 in type II.^5^

## Financial support

None declared.

## Authors' contributions

Evelyn Freitas Rodrigues: Statistical analysis; approval of the final version of the manuscript, design and planning of the study; drafting and editing of the manuscript; collection, analysis, and interpretation of data; effective participation in research orientation; intellectual participation in the propaedeutic and/or therapeutic conduct of the studied cases; critical review of the literature; critical review of the manuscript.

Lisa Gava Baeninger: Statistical Analysis; approval of the final version of the manuscript; design and planning of the study; drafting and editing of the manuscript; collection, analysis, and interpretation of data; effective participation in research orientation; intellectual participation in the propaedeutic and/or therapeutic conduct of the studied cases; critical review of the literature; critical review of the manuscript.

Caroline Romanelli: Statistical analysis; approval of the final version of the manuscript; design and planning of the study; drafting and editing of the manuscript; collection, analysis, and interpretation of data; effective participation in research orientation; intellectual participation in the propaedeutic and/or therapeutic conduct of the studied cases; critical review of the literature; critical review of the manuscript.

## Conflicts of interest

None declared.
